# A Flexible Ultrasound Array for Local Pulse Wave Velocity Monitoring

**DOI:** 10.3390/bios12070479

**Published:** 2022-06-30

**Authors:** Lirui Xu, Peng Wang, Pan Xia, Pang Wu, Xianxiang Chen, Lidong Du, Jiexin Liu, Ning Xue, Zhen Fang

**Affiliations:** 1 Aerospace Information Research Institute, Chinese Academy of Sciences (AIRCAS), Beijing 100190, China; xulirui19@mails.ucas.ac.cn (L.X.); wangpeng01@aircas.ac.cn (P.W.); xiapan17@mails.ucas.ac.cn (P.X.); wupang17@mails.ucas.ac.cn (P.W.); chenxx@aircas.ac.cn (X.C.); 2 School of Electronic, Electrical and Communication Engineering, University of Chinese Academy of Sciences, Beijing 100190, China; 3 Beijing Tiantan Hospital, Capital Medical University, Beijing 100070, China; 4 Personalized Management of Chronic Respiratory Disease, Chinese Academy of Medical Sciences, Beijing 100190, China

**Keywords:** flexible ultrasound array, arterial stiffness, pulse wave velocity, local PWV

## Abstract

Pulse wave velocity (PWV) measured at a specific artery location is called local PWV, which provides the elastic characteristics of arteries and indicates the degree of arterial stiffness. However, the large and cumbersome ultrasound probes require an appropriate sensor position and pressure maintenance, introducing usability constraints. In this paper, we developed a light (0.5 g) and thin (400 μm) flexible ultrasound array by encapsulating 1–3 composite piezoelectric transducers with a silicone elastomer. It can capture the distension waveforms of four arterial positions with a spacing of 10 mm and calculate the local PWV by multi-point fitting. This is illustrated by in vivo experiments, where the local PWV value of five normal subjects ranged from 3.07 to 4.82 m/s, in agreement with earlier studies. The beat-to-beat coefficient of variation (CV) is 12.0% ± 3.5%, showing high reliability. High reproducibility is shown by the results of two groups of independent measurements of three subjects (the error between the mean values is less than 0.3 m/s). These properties of the developed flexible ultrasound array enable the bandage-like application of local PWV monitoring to skin surfaces.

## 1. Introduction

Pulse wave velocity (PWV), the transit velocity of blood waves through the arterial tree, is directly affected by the elastic characteristics of the artery and can be used as an essential indicator for the detection of arterial stiffness [1,2]. The development of PWV measurement is of great importance for the early diagnosis and effective prevention of arterial stiffness. Typically, PWV is measured by pulse transit time (PTT) and the distance from the blood-pulse waveform of two distant superficial artery sites (carotid-femoral, brachial-ankle, etc.). However, three drawbacks of this PWV impede it from clinical application. Firstly, a negative impact on PTT measurement is introduced by wave reflections from arterial bifurcations. Secondly, the accurate measurement of the distance between two distant recording sites is impacted by the irregular geometry of the artery. Normally, the distance measured at the body surface will result in an overestimation of pulse wave velocity [3,4,5]. Finally, arterial mechanical properties differ in different locations, leading to significant differences in PWV across locations [6,7]. It is fortunate that a local PWV method, which is measured at a specific location, can eliminate the influence of waveform reflection and the errors of distance estimation.

In the past few decades, various techniques for the non-invasive assessment of local PWV have been proposed. Among them, the skin-surface PTT-based methods are representative and capture the pulse waveform from superficial arteries over a small segment (typically <5 cm) through photoplethysmographic (PPG) [8,9], pressure [10,11], strain [12,13], magnetic [14,15], and accelerometric [16,17] signals, among others. However, the uneven distance from the artery to the skin surface and the inconsistent time of transmission from the artery to different sensors (tissue transit time) result in an inaccurate estimation of local PWV [11].

Due to the ability of gathering information of arterial pulsations and blood flow by penetrating the human tissue, in recent years, ultrasound signals have been widely used to assess local PWV as cost-effective, non-radioactive approaches (compared to CT and MRI), such as the flow-area loop method [18,19,20] and the PTT method [21,22,23]. The flow-area loop method computes local PWV by simultaneously obtaining the blood flow velocity and arterial cross-sectional area waveforms of a specific artery in a cardiac cycle. The PTT method relies on the time delay estimations of arterial diameter waveforms or blood flow velocity waveforms captured at locations close to each other. Due to the advantages of a simple principle and robust reproducibility, the PTT method is considered to be the preferred method for local PWV measurement. Unfortunately, the large and cumbersome ultrasound probes should be established at an appropriate location and angle with an optimal hold-down pressure. It will inevitably alter artery properties and result in erroneous local PWV measurements [24]. The development of MEMS technology and flexible electronic technology enables a possible solution to these problems. Some miniaturized ultrasound probes [25,26,27] and stretchable ultrasound wearable devices have been developed for blood pressure and blood flow velocity monitoring [24,28,29]. However, there is no research on flexible ultrasound devices for local PWV monitoring.

In this work, we present a thin, lightweight, and flexible ultrasound array for local PWV monitoring. The miniatured ultrasound probe is realized by encapsulating 1–3 composite piezoelectric transducers with a silicone elastomer. The thin and lightweight structure enables the bandage-like application of an ultrasound device to skin surfaces. Local PWV is accurately calculated by linear regression between transmission times and different positions with arrayed ultrasound transducers. It is demonstrated that the developed flexible ultrasound array can be used for local PWV monitoring in vivo.

## 2. Materials and Methods

### 2.1. Measurement Principle and Device Design

**Figure 1:**
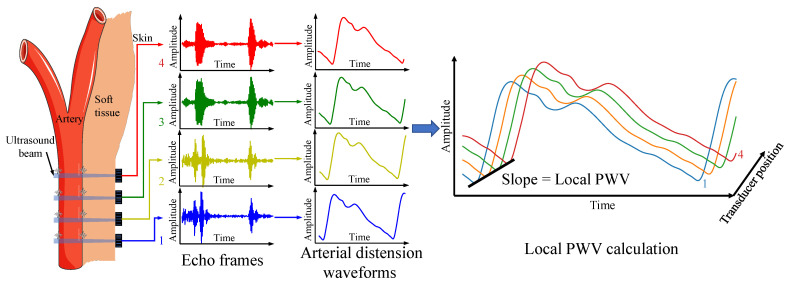
Local PWV measurement principle.

The PTT-based local PWV measurement relies on the time delay of the pulse wave signal from the adjacent position of the artery. When pulsed pressure waves transmit in elastic arteries, proximal artery dilation occurs earlier than the distal artery. The distension waveforms at different arterial locations can be used to calculate local PWV. In this work, multiple ultrasound transducers capture continuous distension waveforms at adjacent arterial positions using the time-of-flight method and calculate the pulse wave velocity using the time–distance linear regression method. Figure 1 illustrates the measurement principle.

**Figure 2:**
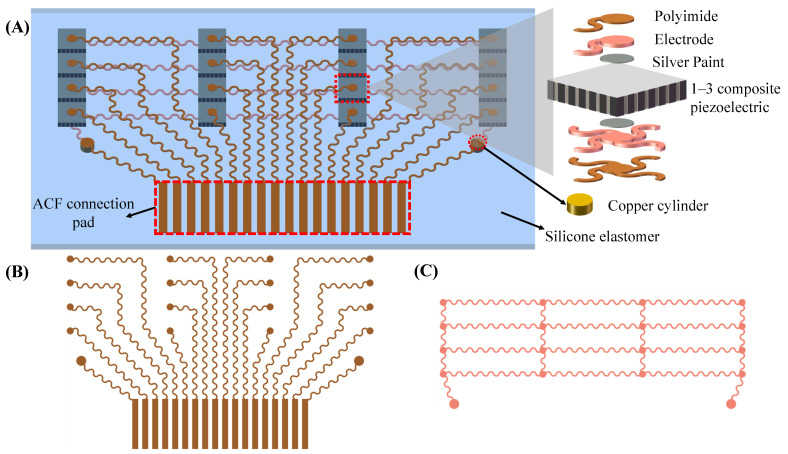
Flexible ultrasound array design and working principle. (**A**) Schematic (left) and exploded view (right) of the device structure. (**B**) Top electrode (polyimide side). (**C**) Bottom electrode (Cu side).

The flexible ultrasound array in this work measures four arterial positions with a spacing of 10 mm for local PWV calculation, so that the final length of the device is only about 30 mm, and the device can be easily attached to the skin. Four transducers were placed at each measurement point, spaced 0.4 mm apart, which helps to cover the underlying artery, ensure that the ultrasound beam passes through the artery, and avoid high operating requirements. The schematic structure of the device is shown in Figure 2A. The device consists of a 4 × 4 array of 1–3 composite piezoelectric transducers, stretchable electrodes (up and bottom), and a silicone package. The 1–3 composite piezoelectric transducer with a center frequency of 5 MHz and a size of 2 by 2 mm can well penetrate tissue to detect deep arteries (see Section 3.1). Compared with traditional piezoelectric materials, a 1–3 composite piezoelectric transducer filled with a polymer matrix between piezoelectric ceramics can effectively suppress shear vibration and improve the longitudinal coupling coefficient [30]. At the same time, due to the decrease in density, the acoustic impedance can be effectively reduced (13.6 megarayleigh in this work). Thus, the 1–3 composite piezoelectric transducer can achieve better acoustic impedance matching with silicone and human tissues, reducing acoustic losses at the interfaces. A 5 μm thick polyimide (PI) and a 20 μm thick Cu stack form a stretchable electrode (Figure 2B,C). The upper electrodes connect each transducer and the anisotropic conductive film (ACF) connector pin separately. The bottom electrode is a common ground electrode, which is connected to the ACF connector of the upper layer through a copper cylinder (Figure 2A bottom right). Each transducer is connected to the top exclusive electrode and the bottom common ground electrode through silver conductive paint.

### 2.2. Fabrication of Flexible Ultrasound Array

#### 2.2.1. Flexible and Stretchable Electrode Patterning

**Figure 3:**
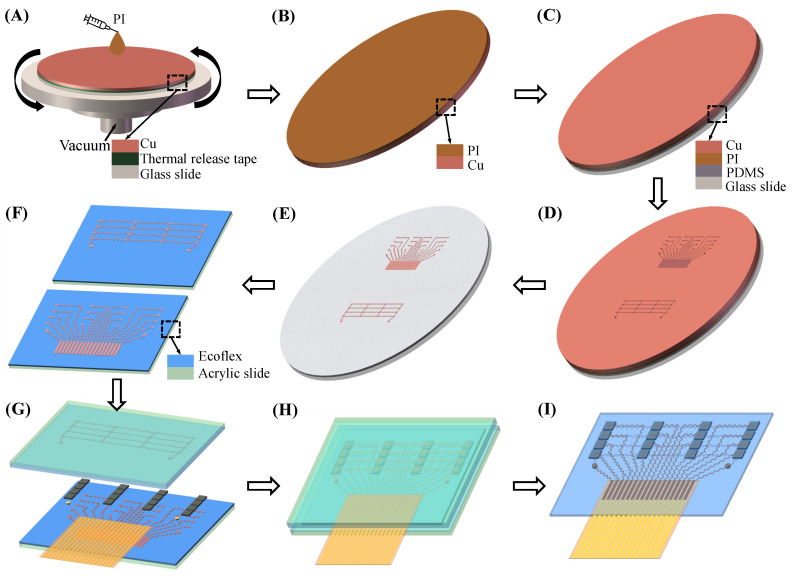
Fabrication process of a flexible ultrasound array (**A**) Spin-coating PI. (**B**) Heat to cure PI and remove the thermal release tape. (**C**) Laminate the PI layer of Cu–PI in contact with the PDMS. (**D**) Stretchable electrodes patterning using the laser. (**E**) The electrode pattern is retained after the excess Cu–PI layer is peeled off. (**F**) The electrode is transferred to the acrylic slide spin-coated with Ecoflex. (**G**) Integration. (**H**) Encapsulated with Ecoflex. (**I**) Acrylic slide removal.

Figure 3 illustrates the fabrication process of the flexible ultrasound array. First, a Cu foil with a thickness of 20 μm was pasted on the glass slide by thermal release tape (release temperature of 150 °C, Qiandingli Dianzi Technology Co., Suzhou, China). PI was spun onto the Cu to a thickness of 10 μm. The PI layer was cured at 80 °C for 10 min, 120 °C for 10 min, 150 °C for 10 min (the thermal release tape was at this point removed), 180 °C for 40 min, and 210 °C for 1 h. A glass slide spin-coated with PDMS (a Sylgard 184 silicone elastomer, bases and curing agent at a 10:1 ratio) served as a temporary substrate to laminate the PI layer of Cu–PI in contact with the PDMS. The Cu–PI was patterned into the shape of a stretchable conduct wire (top and bottom electrodes) by a laser. After the excess Cu–PI layer was peeled off, the electrodes left on the substrate were cleaned with 70% acetic acid to remove the oxide layer.

#### 2.2.2. Integration and Packaging

The Cu–PI electrode was transferred to the acrylic slide spin-coated with Ecoflex (00-30, Smooth-On) by water-soluble tape (9969B, 3M). Using ACF tape (7303, 3M), a flexible flat cable was connected to the ACF connection pad of the upper electrode. Top and bottom electrodes, 16 1–3 composite piezoelectric transducers, and two copper cylinders were then bonded together with conductive silver paste, and they were cured at 75 °C for 1 h. Next, they were encapsulated with Ecoflex under negative pressure to remove gaps. After Ecoflex was cured at room temperature for 3 h, and the acrylic slide and excess Ecoflex were peeled off. Finally, medical silicone adhesive was applied to the bottom of the flexible ultrasound array so that it can adhere tightly to the skin, eliminating gaps to avoid the use of gels.

### 2.3. Data Acquisition Hardware

**Figure 4:**
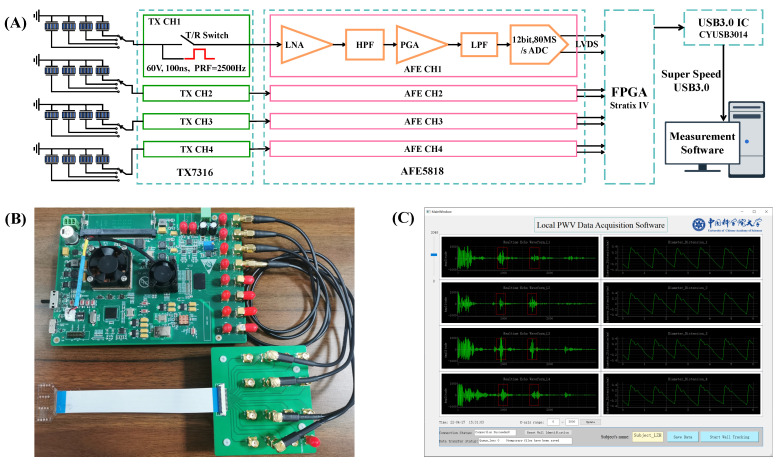
Data acquisition system. (**A**) Multi-channel ultrasound system hardware architecture. (**B**) Electronics hardware board. (**C**) A screenshot of the software graphical user interface (GUI).

A custom-integrated multi-channel ultrasound system was designed for transmitting, receiving, and pre-processing ultrasound signals. The hardware architecture and electronics hardware board are illustrated in Figure 4A,B. In this hardware system, an Altera Stratix IV FPGA (Intel, Santa Clara, CA, USA) was used to generate high-speed digital pulses with a duration of 100 ns and a pulse repetition rate of 2500 Hz. The digital pulses were fed to a 16-channel high voltage pulse transmitter (TX7316, Texas Instruments, Dallas, TX, USA). The TX7316 performed level conversion to generate a 60 V high voltage pulse to excite the ultrasound transducers. After sending the high-voltage excitation, the transmit/receive (T/R) switch inside TX7316 was switched to the receiving mode, and the low-voltage echo signal was transmitted to the 16-channel ultrasound special analog front end (AFE5818, Texas Instruments, Dallas, TX, USA). In AFE818, the echo signal was amplified by a low-noise amplifier (LNA) and programmable gain amplifier (PGA) for a total of 48 dB and passed through a high pass filter with a cut-off frequency of 200 kHz and a low-pass filter with a cut-off frequency of 10M Hz. The pre-processed signal was digitized by the integrated 80 Msps, 12-bit ADC, and transferred to the FPGA by low-voltage differential signaling (LVDS). The FPGA receives the digital signals from the LVDS and transmits them from the super-speed USB3.0 chip (CYUSB3014, Infineon, Neubiberg, Germany) to the PC.

The ultrasound array was interfaced to the hardware through four 4-line to 1-line channel selectors. In each column of the array, a channel whose sound field was close to the arterial diameter (the echo of the proximal and distal walls was strong, and the software can identify them and calculate the arterial distension waveforms in real time) was selected into the hardware board. Thus, only four channels of the hardware system were used. To ensure the synchronization of the four channels, the FPGA controlled the four channels of TX7316 to send ultrasound excitation at the same time. The timing of the T/R switch, ultrasound excitation, and ADC acquisition was as follows: First, after the T/R switch was turned off for 2 μs, a square wave excitation signal with a width of 100 ns was emitted to the transducer, and the closed T/R switch avoided the high voltage excitation access to the ADC. Next, after emitting the excitation for 1 μs, the T/R switch was turned on. Finally, after 2 μs of excitation, ADC captured echo signals for 37.5 μs (3000 sampling points), which means that tissue echoes with a depth range of 1.54–30.42 mm were captured (considering a 1540 m/s speed for the sound in human tissue).

### 2.4. Measurement Software

The calculation of the arterial distension waveform from echo frames involves many cross-correlation calculations [31]. The operation of calculating local PWV from 2500 frames per second requires offline processing. A special graphical user interface (GUI) designed with Python and PYQT5 in a Windows environment was developed for the echo data display and storage. The measurement software was composed of two parts: an online low-frame-rate data quality evaluation and display, and an offline high-frame-rate, high-precision local PWV calculation. In the low-frame-rate mode, the data of 2500 frames per second was extracted and reduced to 50 frames per second. The algorithm automatically identified the arterial wall locations according to the relative displacement relationship between the proximal wall and distal wall of the artery [32]. Once the arterial wall position was identified, the cross-correlation wall tracking algorithm was used to calculate the arterial distension waveform. The echo waveform and arterial distension waveform were displayed in the GUI (Figure 4C) in real time. The operator can adjust the pasting position of the device or select an appropriate channel according to the signal quality and decide whether to save the high-frame-rate data.

The stored four channels’ high-frame-rate data was used for offline local PWV calculation. First, the original frames were filtered using a 4th order finite impulse response (FIR) band-pass filter with cut-off frequencies of 2 MHz and 8 MHz. The arterial wall auto-identification algorithm [32] was applied to locate the proximal and distal walls of the arteries. The positions at 1 mm in front of and behind the proximal and distal walls were selected as the regions of interest (ROIs) for tracing arterial distension. Since echo frames were acquired at a high frame rate, the resolution of tracking should be improved accordingly. The ROIs of the frames were interpolated to 1.6 GHz and applied to the wall tracking algorithm to obtain high-resolution arterial distension waveforms [31].

The four channels’ arterial distension waveforms were further filtered by a 3rd order zero phase shift band-pass filter with cut-off frequencies of 0.5 Hz and 20 Hz. Subsequently, the filtered waveforms were interpolated to 10 kHz. In this way, the resolution of the PTT could reach 0.1 ms. The interpolated waveforms were then used to calculate the characteristic points of the PTT. The second derivative maximum of the waveform was selected as the time reference point, because it is considered not to be affected by the reflection of the waveform and can provide high repeatability [8,33]. Finally, the local PWV was calculated by linear regression between four beam positions and transmission times.

### 2.5. In Vivo Experiments

The in vivo experiment was carried out on five subjects to verify the functions of the proposed flexible ultrasound array and measurement system. The subjects included 4 men and 1 woman with a normal blood pressure (age = 26.2 ± 1.3 years and body mass index = 21.6 ± 2.0 kg/m${}^{2}$). The carotid artery was the preferred measurement location because it is closely connected to the aorta and provides a straight, bifurcation-free path. The measurement was performed by an operator in the supine position of the subject. Before the measurement, the subject rested for 5 min to ensure hemodynamic stability. By adjusting the position of the sensor and selecting the appropriate data channel through the channel selectors until the high-quality arterial echo signal was obtained at least at 3 positions, the operator saved the echo data of 15 s for calculating and analyzing the local PWV beat by beat (Trial 1). In order to evaluate the reproducibility of the device, two independent experiments were conducted on 3 subjects. The subjects continued to maintain the same posture, and the operator pasted the sensor in the same position to ensure that the physiological conditions of the two experiments were similar. The echo data of 15 s was also saved to analyze reproducibility (Trial 2).

## 3. Results and Discussions

### 3.1. Device Characterization

**Figure 5:**
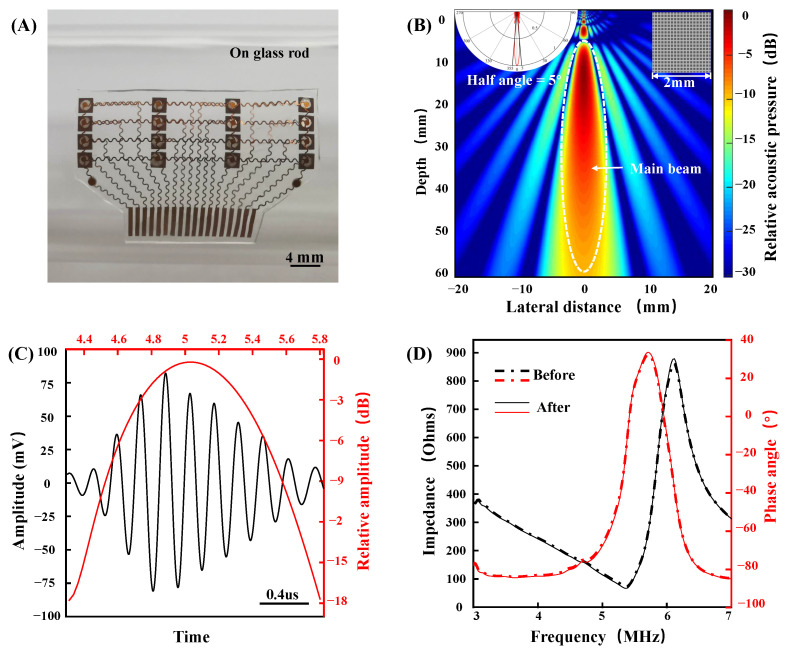
Device characterization. (**A**) Optical image of the device when bent around a curved glass rod. (**B**) Theoretical beam pattern of a single 1–3 composite piezoelectric transducer. (**C**) Time and frequency domain characterizations of the signal from a carotid arterial distal wall. (**D**) The impedance and phase angle of the device before and after 300 bending cycles.

The fabricated device has a size of 34 by 22 mm, a thickness of 0.4 mm, and a weight of only 0.5 g. The device can be well combined with the curved surface to ensure conformal contact with the skin. Figure 5A shows the optical image of the fabricated device when bent around a curved glass rod. The device has the characteristics of a small volume, a light weight, and conformability with skin, increasing its potential for skin integration applications. Figure 5B shows the theoretical beam pattern of a single 1–3 composite piezoelectric transducer (MATLAB r2019a, translator array calculation GUI). The directivity pattern indicates that the half angle of the sound beam is only 5.0°, showing good beam directivity, which ensures that the transducer signals in each column do not interfere with each other. The penetration depth is more than 5 cm, which can be used for the detection of a deep artery (such as a carotid artery). The time domain and frequency domain analysis of carotid arterial distal wall echo signal from a healthy volunteer is shown in Figure 5C. The echo center frequency is approximately 5 MHz. For flexible devices that are attached to the surface of the skin, stable performance is of paramount importance. We tested the impedance and phase angle of the device before and after 300 bending cycles (Figure 5D). The impedance and phase angle of piezoelectric material reflects its electromechanical conversion efficiency. The result shows that the performance of piezoelectric materials is consistent before and after bending.

### 3.2. Device Performance of In Vivo Experiments

**Figure 6:**
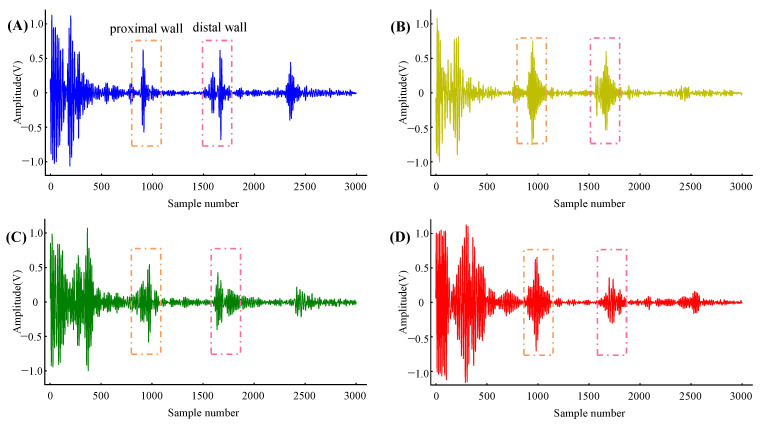
Echo frames captured from four arterial positions with a spacing of 10 mm. (**A**–**D**) are from Column 1, Column 2, Column 3, and Column 4, respectively.

The local PWV was calculated from the distension waveform of the artery diameter captured from the multi-channel echo frames. Figure 6 shows the four channels’ echo frame obtained synchronously in the carotid artery. Typically, echo frames of carotid arterial wall echoes contain 3 to 4 echogenic envelopes originating from various tissue interfaces (due to changes in acoustic impedance). The first envelope is the echo from the superficial tissue, which has a strong amplitude due to less attenuation in the area close to the transducer. The following two envelopes are from the proximal and distal walls of the carotid artery, and they move in opposite directions between consecutive frames. The fourth envelope is static noise, which originates from a fixed tissue structure and is usually time-invariant. The fourth envelope’s morphology varies depending on the subject and where it is measured; in some cases, it may not be present. After processing the cross-correlation between the proximal and distal wall regions in the continuous echo frames, the continuous distension waveforms of the artery diameter were obtained.

**Figure 7:**
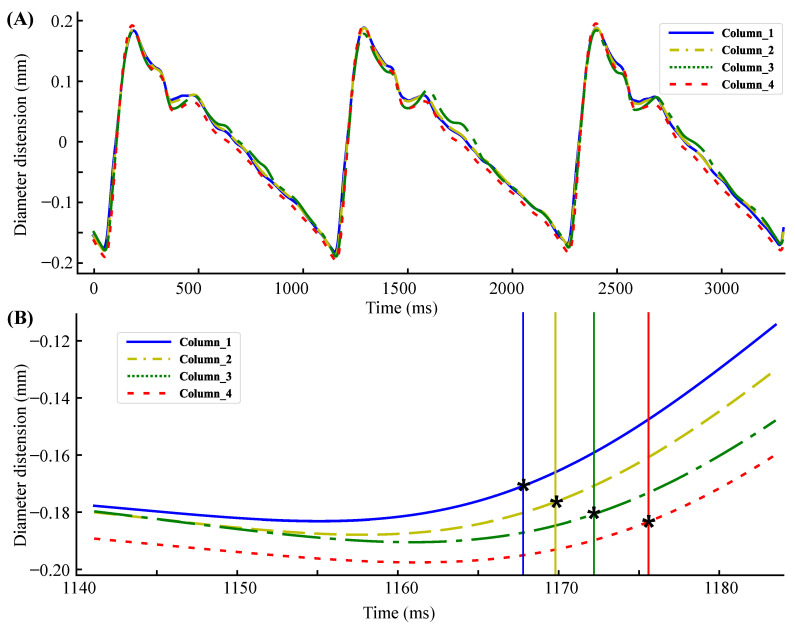
(**A**) Carotid aretery diameter distension waveforms of three cardiac cycles. (**B**) Delay details of the diameter distension waveforms. * represents the maximum point of the second derivative.

The carotid artery diameter distension waveforms of three cardiac cycles obtained from a subject are shown in Figure 7A. The diameter changes of the four locations in Figure 7A are not entirely the same because the relative position relationship between the ultrasound beam and the diameter is different. The closer the ultrasound beam is to the center of the artery, the greater the diameter changes are. Since the dilatation of the carotid artery is uniform in all directions, this does not affect the judgment of time delay. The second derivative maximum point of the diameter distension waveform was used as the time reference point. Figure 7B shows the delay details of the diameter distension waveforms.

**Figure 8:**
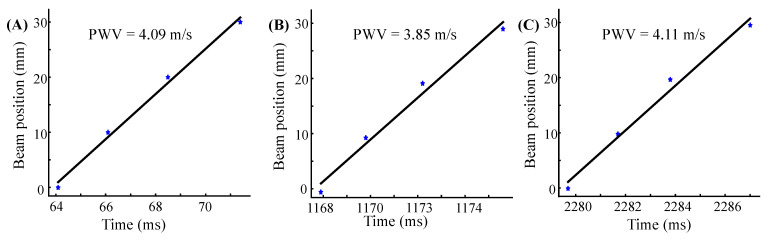
Time reference points (blue star) plotted as a function of transducer positions as well as linear fitting lines (black line). (**A**–**C**) were measured from three consecutive cardiac cycles of subject 2 (Trial 2).

In the hardware, the data were obtained with a frame rate of 2.5 KHz and was interpolated to 10 KHz by the software, ensuring the resolution of the time reference point to 0.1 ms. Figure 8 shows the linear fitting line of the same subject in three consecutive cardiac cycles. The horizontal and vertical coordinates of the graph are the time and beam position, respectively, and the slope of the fitting line is the local PWV. The calculated local PWV is 4.09 m/s, 3.85 m/s, and 4.11 m/s, respectively.

**Figure 9:**
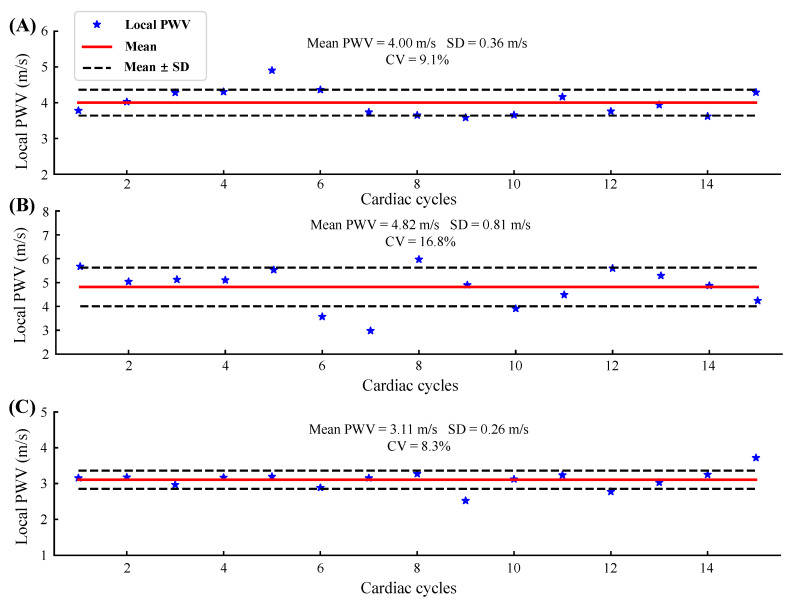
Beat-to-beat local PWV for 15 cardiac cycles of three subjects: (**A**) S1, Trial 1; (**B**) S4, Trial 2; (**C**) S5, Trial 2.

Figure 9 shows the beat-to-beat local PWV for 15 cardiac cycles of three subjects. The solid red line and the dotted black line are the mean and the mean ± standard deviation (SD) of the local PWV, respectively. The coefficient of variation (CV) was obtained by dividing the SD by the mean, reflecting the measurement reliability. Table 1 shows the statistical results of the local PWV measurements in five subjects. The table also records the transducers used in the experiment, which are represented by column numbers and row numbers.

The absolute value of the local PWV obtained in five normal subjects ranged from 3.07 to 4.82 m/s, and the average value was 3.9 ± 0.66 m/s, which was close to that of previous studies [8,34,35]. The CV ranges from 8.3% to 16.8% (12.1% ± 3.4%). Since there are no previous studies related to local PWV measured using flexible ultrasound arrays, we are unable to compare it with similar work. In the study of the carotid artery local PWV using rigid ultrasound probes, the authors in [34] used an imaging probe, and the CV of the local PWV obtained ranged from 7% to 11%; the authors in [35] used a custom-designed dual element ultrasound probe, and the CV of the measured local PWV was less than 5%. The CV of the local PWV measured in this study was slightly larger than that of the rigid probe, which is acceptable for small flexible devices. Furthermore, for the arterial mechanics state assessment, the problem of a large beat-by-beat CV can be overcome by averaging over multiple cardiac cycles [20].

### 3.3. Study Limitations and Future Research Directions

We have thoroughly investigated the reliability and reproducibility of the proposed flexible ultrasound array in measuring local PWV in vivo. However, because there is currently no clinically available gold standard for non-invasively measuring local PWV, the accuracy in the application in clinical settings is ambiguous. In future studies, invasive arterial catheter techniques are required to obtain a reference for the actual value of the local PWV. In addition, it is often necessary to adjust the position of the sensor several times in in vivo experiments to obtain high-quality arterial echo signals. Although the spacing between each row of the array is only 0.4 mm to cover the arterial vessels, since the arteries are circular, strong echoes can only be obtained when the ultrasound beam is perpendicular to the artery and aligned with the diameter. Finally, the body movement significantly interferes with the local PWV measurement, which is a common problem in research on flexible ultrasound arrays [24,28,29]. For example, wider transducers can be used to ensure that the sound beam covers the lower artery, to reduce the interference caused by motion, and to cancel the requirement of a pasted position. However, appropriate algorithms should be used to improve the low signal-to-noise ratio caused by the tissue echo noise around the artery.

## 4. Conclusions

In this study, we developed a light, thin (4 × 4), flexible ultrasound array by encapsulating 1–3 composite piezoelectric transducers with a silicone elastomer. It can capture the distension waveforms of four arterial positions with a spacing of 10 mm and calculate the local PWV by multi-point fitting. At the same time, a complete measurement system was developed for data acquisition, including multi-channel, integrated, high-speed ultrasound transceiver hardware and software for data reception, display, and processing. It is illustrated by in vivo experiments that the local PWV value of five normal subjects ranged from 3.1 to 4.8 m/s, in agreement with earlier studies.The beat-to-beat coefficient of variation is 12.0% ± 3.5%, showing high reliability. High reproducibility is shown by the results of two groups of independent measurements of three subjects (the error between the mean values is less than 0.3 m/s). These properties of the developed flexible ultrasound array enable the bandage-like application of an ultrasound device on skin surfaces and improve upon the traditional ultrasound local PWV sensors.

## Figures and Tables

**Table 1:** Statistical results of the local PWV measurements in five subjects.

Subject ID	Age (Years)	Trial ID	Elements Used		Mean ± SD ( m/s)	CV
			Column Numbers	Row Numbers		
S1	26	1	1, 2, 3, 4	2	4.00 ± 0.36	9.1%
S2	24	1	2, 3, 4	2	3.07 ± 0.49	16.1%
S3	26	1	1, 2, 3, 4	2	4.43 ± 0.68	15.5%
		2	1, 2, 3, 4	3	4.11 ± 0.37	9.0%
S4	28	1	2, 3, 4	1	4.60 ± 0.60	13.0%
		2	1, 2, 3	2	4.82 ± 0.81	16.8%
S5	26	1	1, 2, 3, 4	3	3.23 ± 0.28	8.6%
		2	1, 2, 3, 4	3	3.11 ± 0.26	8.3%

## Data Availability

The data presented in this study are available from the author upon request.

## References

[1] Nichols W.W. (2005). Clinical measurement of arterial stiffness obtained from noninvasive pressure waveforms. Am. J. Hypertens..

[2] Ben-Shlomo Y., Spears M., Boustred C., May M., Anderson S.G., Benjamin E.J., Boutouyrie P., Cameron J., Chen C.-H., Cruickshank J.K. (2014). Aortic pulse wave velocity improves cardiovascular event prediction: An individual participant meta-analysis of prospective observational data from 17,635 subjects. J. Am. Coll. Cardiol..

[3] Rajzer M.W., Wojciechowska W., Klocek M., Palka I., Brzozowska-Kiszka M., Kawecka-Jaszcz K. (2008). Comparison of aortic pulse wave velocity measured by three techniques: Complior, SphygmoCor and Arteriograph. J. Hypertens..

[4] Hirata K., Kawakami M., O’Rourke M.F. (2006). Pulse Wave Analysis and Pulse Wave Velocity. Circ. J..

[5] Boutouyrie P., Briet M., Collin C., Vermeersch S., Pannier B. (2009). Assessment of pulse wave velocity. Artery Res..

[6] Luo J., Fujikura K., Tyrie L.S., Tilson M.D., Konofagou E.E. (2009). Pulse wave imaging of normal and aneurysmal abdominal aortas in vivo. IEEE Trans. Med. Imaging.

[7] Baguet J.P., Kingwell B.A., Dart A.L., Shaw J., Ferrier K.E., Jennings G.L. (2003). Analysis of the regional pulse wave velocity by Doppler: Methodology and reproducibility. J. Hum. Hypertens..

[8] Nabeel P.M., Karthik S., Joseph J., Sivaprakasam M. (2018). Arterial Blood Pressure Estimation From Local Pulse Wave Velocity Using Dual-Element Photoplethysmograph Probe. IEEE Trans. Instrum. Meas..

[9] Pereira T., Santos I., Oliveira T. (2013). Characterization of Optical System for Hemodynamic Multi-Parameter Assessment. Cardiovasc. Eng. Technol..

[10] Pereira H.C., Pereira T., Almeida V., Borges E., Figueiras E., Simões J.B., Malaquias J.L., Cardoso J.M., Correia C.M. (2010). Characterization of a double probe for local pulse wave velocity assessment. Physiol. Meas..

[11] Hsu Y., Young D.J. (2014). Skin-Coupled Personal Wearable Ambulatory Pulse Wave Velocity Monitoring System Using Microelectromechanical Sensors. IEEE Sens. J..

[12] Rachim V.P., Kang S., Baek J.H., Park S.M. (2021). Unobtrusive, Cuffless Blood Pressure Monitoring Using a Soft Polymer Sensor Array With Flexible Hybrid Electronics. IEEE Sens. J..

[13] Shu Y., Li C., Wang Z., Mi W., Li Y., Ren T.-L. (2015). A Pressure Sensing System for Heart Rate Monitoring with Polymer-Based Pressure Sensors and An Anti-Interference Post Processing Circuit. Sensors.

[14] Nabeel P.M., Joseph J., Sivaprakasam M. Arterial compliance probe for local blood pulse wave velocity measurement. Proceedings of the 2015 37th Annual International Conference of the IEEE Engineering in Medicine and Biology Society (EMBC).

[15] Lee W.H., Rho Y.H. (2016). Measurement of Cuffless Blood Pressure by Using a Magnetoplethysmogram Pulsimeter. Insights Blood Press..

[16] Lascio N.D., Bruno R.M., Stea F., Bianchini E., Gemignani V., Ghiadoni L., Faita F. (2014). Non-invasive assessment of carotid PWV via accelerometric sensors: Validation of a new device and comparison with established techniques. Eur. J. Appl. Physiol..

[17] Arathy R., Nabeel P.M., Joseph J., Sivaprakasam M. (2019). Accelerometric patch probe for cuffless blood pressure evaluation from carotid local pulse wave velocity: Design, development, and in vivo experimental study. Biomed. Phys. Eng. Express.

[18] Rabben S.I., Stergiopulos N., Hellevik L.R., Smiseth O.A., Slørdahl S., Urheim S., Angelsen B. (2004). An ultrasound-based method for determining pulse wave velocity in superficial arteries. J. Biomech..

[19] Segers P., Swillens A., Taelman L., Vierendeels J. (2014). Wave reflection leads to over- and underestimation of local wave speed by the PU- and QA-loop methods: Theoretical basis and solution to the problem. Physiol. Meas..

[20] Seo J., Pietrangelo S.J., Sodini C.G., Lee H.S. (2018). Motion Tolerant Unfocused Imaging of Physiological Waveforms for Blood Pressure Waveform Estimation Using Ultrasound. IEEE Trans. Ultrason. Ferroelectr. Freq. Control..

[21] Meinders J.M., Kornet L., Brands P.J., Hoeks A.P. (2001). Assessment of local pulse wave velocity in arteries using 2D distension waveforms. Ultrason. Imaging.

[22] Deng L., Zhang Y., Chen Z., Zhao Z., Zhang K., Wu J. (2020). Regional Upstroke Tracking for Transit Time Detection to Improve the Ultrasound-Based Local PWV Estimation in Carotid Arteries. IEEE Trans. Ultrason. Ferroelectr. Freq. Control.

[23] Eriksson A., Greiff E., Loupas T., Persson M., Pesque P. (2002). Arterial pulse wave velocity with tissue doppler imaging. Ultrasound Med. Biol..

[24] Wang C.H., Li X.S., Hu H.J., Zhang L., Huang Z.L., Lin M.Y., Zhang Z.R., Yin Z.N., Huang B., Gong H. (2018). Monitoring of the central blood pressure waveform via a conformal ultrasonic device. Nat. Biomed. Eng..

[25] Yang Y., Tian H., Yan B., Sun H., Wu C., Shu Y., Wang L.-G., Ren T.-L. (2013). A flexible piezoelectric micromachined ultrasound transducer. RSC Adv..

[26] Hu H.J., Zhu X., Wang C.H., Zhang L., Li X.S., Lee S., Huang Z.L., Chen R.M., Chen Z.Y., Wang C.F. (2018). Stretchable ultrasonic transducer arrays for three-dimensional imaging on complex surfaces. Sci. Adv..

[27] Sun S., Zhang M., Gao C., Liu B., Pang W. Flexible piezoelectric micromachined ultrasonic transducers towards new applications. Proceedings of the 2018 IEEE International Ultrasonics Symposium (IUS).

[28] Wang C.H., Qi B.Y., Lin M.Y., Zhang Z.R., Makihata M., Liu B.Y., Zhou S., Huang Y.H., Hu H.J., Gu Y. (2021). Continuous monitoring of deep-tissue haemodynamics with stretchable ultrasonic phased arrays. Nat. Biomed. Eng..

[29] Wang F.L., Jin P., Feng Y.L., Fu J., Wang P., Liu X., Zhang Y.C., Ma Y.J., Yang Y.Y., Yang A.M. (2021). Flexible Doppler ultrasound device for the monitoring of blood flow velocity. Sci. Adv..

[30] Smith W.A., Auld B.A. (1991). Modeling 1–3 composite piezoelectrics: Thickness-mode oscillations. IEEE Trans. Ultrason. Ferroelectr. Freq. Control..

[31] Brands P.J., Hoeks A.P.G., Ledoux L.A.F., Reneman R.S. (1997). A radio frequency domain complex cross-correlation model to estimate blood flow velocity and tissue motion by means of ultrasound. Ultrasound Med. Biol..

[32] Sahani A.K., Joseph J., Sivaprakasam M. (2014). Evaluation of the algorithm for automatic identification of the common carotid artery in ARTSENS. Physiol. Meas..

[33] Mukkamala R., Hahn J.O., Inan O.T., Mestha L.K., Kim C.S., Toreyin H., Kyal S. (2015). Toward Ubiquitous Blood Pressure Monitoring via Pulse Transit Time: Theory and Practice. IEEE Trans. Biomed. Eng..

[34] Luo J., Li R.X., Konofagou E.E. (2012). Pulse wave imaging of the human carotid artery: An in vivo feasibility study. IEEE Trans. Ultrason. Ferroelectr. Freq. Control..

[35] Kiran V.R., Nabeel P.M., Joseph J. (2022). Image-Free Fast Ultrasound for Measurement of Local Pulse Wave Velocity: In vitro Validation and In vivo Feasibility. IEEE Trans. Ultrason. Ferroelectr. Freq. Control..

